# Supplementary material for: Age-related differences in driving behaviors among non-professional drivers in Egypt
Source: PLoS One. 2020 Sep 3;15(9):e0238516. doi: 10.1371/journal.pone.0238516 (PMC7470271; doi:10.1371/journal.pone.0238516)
Supplement: Attachment — Submitted filename: Driving Response (PLOS).docx [file pone.0238516.s002.docx]

Dear Editor in Chief,

Thank you for the valuable comments.

Herein, we included our response to the comments made by the editor and reviewers.

**Editor**

1. Further, the English writing is considerably poor, and my recommendation is to submit the paper to a professional editorial proof-reading.

Response: The manuscript was reviewed by a professional editorial proof-reading as suggested.

2. Also, the introduction of the paper lacks enough background and support for psychosocial risk factors that have been demonstrated to influence/affect profesional driver's risky road behaviors (i.e., stress, fatigue, etc.), and more context on it (even though you did not measure all these variables, but they still count) and discussion in the glance of your results is needed. For this stage, please consider to access original research papers published in high-impact journals, showing empirical data and (if possible) predictive models on the explanation of risky road behavior among Professional Drivers, even if not all the studies have been performed in Egypt.

Response: The introduction was modified as suggested (Page 5,6). We added brief descriptions for studies assessing the relationship between the age of drivers and their driving behaviors and crash involvement. We also added a brief description of the factors affecting driving behaviors (Page 5 lines 19-25 & Page 6 lines 26-36).

**Journal Requirements**

When submitting your revision, we need you to address these additional requirements.

1. Please ensure that your manuscript meets PLOS ONE's style requirements, including those for file naming.

 Response: Modified throughout the manuscript as requested.

2. Please provide additional details regarding participant consent. In the ethics statement in the Methods and online submission information, please ensure that you have specified (1) whether consent was informed and (2) what type you obtained (for instance, written or verbal, and if verbal, how it was documented and witnessed). If your study included minors, state whether you obtained consent from parents or guardians. If the need for consent was waived by the ethics committee, please include this information.

 Response: The ethical considerations section was modified as requested (Page 9 lines 103-107).

3. Please include additional information regarding the survey or questionnaire used in the study and ensure that you have provided sufficient details that others could replicate the analyses. For instance, if you developed a questionnaire as part of this study and it is not under a copyright more restrictive than CC-BY, please include a copy, in both the original language and English, as Supporting Information.

Response: We added more details about the questionnaire (Page 8 lines 82-98 & Page 9 lines 99-102). We also made all data available.

**Reviewers' comments**

Reviewer's Responses to Questions

**Comments to the Author**

 1. Is the manuscript technically sound, and do the data support the conclusions?
Reviewer #1: No
Reviewer #2: Yes

Response: We conducted rigorous modifications to make the manuscript technically clear.

2. Has the statistical analysis been performed appropriately and rigorously?
   Reviewer #1: Yes
   Reviewer #2: Yes

 Response: Thank you.

3. Have the authors made all data underlying the findings in their manuscript fully available?
   Reviewer #1: No
   Reviewer #2: No

Response: We made all data available.

4. Is the manuscript presented in an intelligible fashion and written in standard English?
   Reviewer #1: No
   Reviewer #2: Yes

Response: The manuscript was reviewed by a professional editorial proof-reading.

**Review Comments to the Author**
**Reviewer #1:** This manuscript presents a study of driver behavior by age group among non-professional drivers in an Egyptian city. I have several concerns with the manuscript.

1) The manuscript does not provide a strong rationale for conducting the study. It is already well-documented that young drivers are risk-takers, inexperienced, and sensation seekers and it is already well-documented that older drivers make mistakes while driving due to declining abilities and are not risk takers. Given that this paper focuses on a single city in Egypt, the manuscript should present arguments why it is important to investigate driver behavior in Beni-Suef.

Response: We added an argument about the importance of investigating the behaviors of drivers in Egypt (Page 6 lines 37-46). We made a further argument about why studying the associations with driving behaviors was required in Beni-Suef governorate (Page 7 lines 54-61).

2) The introduction does not show a thorough understanding of the constructs underlying the driving behavior questionnaire (DBQ). Yes, the questionnaire divides aberrant driving behaviors into violations, errors, and lapses, it does so because these behaviors presumably result from different mechanisms: Violations result from risk-taking and errors from declining abilities. Many studies do not even include the lapses construct because it is not clear what underlies this type of behavior. Given the focus on driver age in this study, the authors should generate hypotheses based on these mechanisms.

Response: We agree with you, therefore, we added further descriptions of driving behaviors and their possible associations with age. We also added brief examples of previous studies in this regard. As suggested, we generated a hypothesis postulating that young drivers could have more violations and errors while old drivers could have more lapses (Page 5 lines 19-25 & Page 6 lines 26-36).

3) The DBQ driver behavior questionnaire has been used extensively around the world to study driver behavior by age group. This research should be reviewed briefly in the introduction not in the results section of the paper.

Response: We added a brief review of earlier research investigating the relationship between the age of drivers and their driving behaviors to the introduction section (Page 5 lines 19-25 & Page 6 lines 26-36).

4) The description of the methods is missing critical details. How were specific drivers selected to be in the study? Were they compensated for participation? How was the questionnaire administered (e.g., paper and pencil; Internet)? How was the purported randomization achieved? Given the focus on driver age, why were the three age groups balanced for equal cell sizes? Why weren’t the groups also balanced by gender, since it is know that driver self-report is often influenced by gender?

Response: We added more details to the methods section about the selection and data collection process (Page 8 lines 76-81).

Regarding the possibility of balancing the group by sex, this study was cross-sectional, and the cut-offs of age groups were decided before data collection, thus, drivers were not equally distributed across age groups: 560 young, 850 middle-aged, and 354 old. Besides, because women represent a small portion of non-professional drivers in Egypt and putting in mind that seeing a woman driving in South Egypt 30 years ago was uncommon, it was predicted to have a small number of women in the old age group (≥ 60 years) of drivers. However, to minimize the effect of sex, we adjusted the differences for sex and other variables, and it could be shown in (Table 2) that the adjusted model did not differ significantly from the unadjusted one.

5) The designation of “old driver” as being age 60 or older is counter to the contemporary research and thinking in the older driver literature. Work over the past decade defines an older driver as either 65 years or 70 years and older. Indeed, the most recent work has defined older driver as 75 years or older. The fact that the mean age of the older driver group in this study was less than 65 years of age indicates that this group would not be considered older drivers to other researchers in the field of aging and transportation. This also probably is the reason why no significant differences were found between middle and old drivers on overall errors, lapses, or violations.

Response: While most studies from the USA, Europe, and Japan use 65 years as a cut-off for old age, the Egyptian studies use 60 years as a cut-off. This cut-off is widely used nationwide for many reasons; 1- It is the age of pension in Egypt, 2- It is the age used by the Egyptian Census to refer to old people, 3- It is the age used by the Government to offer social welfare programs for old people, and 4- The overall average of life expectancy in Egypt is 71.5 years compared with 84.5 years in Japan, 83.5 years in Italy, Spain, Switzerland, and Australia, and 79 years in the USA.

- Aly HY, Hamed AF, Mohammed NA. Depression among the elderly population in Sohag governorate. Saudi Med J. 2018;39(2):185‐190.
- Ahmed D, El Shair IH, Taher E, Zyada F. Prevalence and predictors of depression and anxiety among the elderly population living in geriatric homes in Cairo, Egypt. J Egypt Public Health Assoc. 2014 Dec;89(3):127-35.
- Central Agency for Public Mobilization and Statistics (CAPMAS). Egypt statistics. Final results of 2017 Census; <http://www.capmas.gov.eg>

6) The authors should justify why comparisons for young and old drivers were made for middle age drovers, but not between each other. One would expect the greatest differences when comparing young versus old.

Response: Since our primary hypothesis was to detect aberrant driving behaviors in young and old drivers and to test whether young drivers had more violations and errors and old drivers had more lapses, we had to use the middle-aged people as a reference group because in our situation both young and old groups were study groups.

7) The second paragraph of the discussion implies that aberrant and risky driving behaviors are distinct. I would argue that risky driving behavior is one type of aberrant driving behavior and would, in particular, fall under the violations category of the DBQ. The authors should clarify.

Response: We agree with you and we modified this section as suggested (Page 11 line 151).

8) The discussion includes some statements that are not supported by the literature and/or do not include proper citations. For example, the fourth paragraph of the discussion states that old drivers are aware of their cognitive and physical limitations and they therefore tend to exhibit self-monitoring behavior. This statement of fact does not have a citation and it is not supported by the research on older driver self-regulation.

Response: We clarified the citation and we added 2 more citations suggesting self-monitoring behavior among old drivers (Page 12 lines 176-182). However, we agree with you that some sections need proper citations and therefore we updated and added some references.

- Anstey K, Wood J, Lord S, Walker J. Cognitive, sensory and physical factors enabling driving safety in older adults. Clin Psychol Rev, 2005; 25:45-65.
- Conlon E, Rahaley N, Davis J. The influence of age-related health difficulties and attitudes toward driving on driving self-regulation in the baby boomer and older adult generations. Accid Anal Prev, 2017; 102:12-22.
- Molnar L, Eby D, Charlton J, Langford J, Koppel S, Marshall S, et al. Driving avoidance by older adults: is it always self-regulation? Accid Anal Prev, 2013; 57:96-104.

**Reviewer #2**: This study investigates differences in driving behaviour for younger and older drivers (compared to middle-aged drivers) in a large sample of Egyptian drivers. Young drivers report significantly more risk-taking and violations. Differences between middle and older drivers were less common with older drivers showing more cautious driving than middle aged.
1). The comparison groups of <25 and >59 are interesting. I was not sure that “middle-aged” was an appropriate term for the whole 25-59 age group, particularly for 26-39. However, I don’t have an immediate suggestion for a better label for this group.
Response: Defining people <60 years as middle-aged was used before in research.

- Tsugane S, Sasaki S, Tsubono Y. Under- and overweight impact on mortality among middle-aged Japanese men and women: a 10-y follow-up of JPHC study cohort I. Int J Obes Relat Metab Disord. 2002 Apr;26(4):529-37.
- Yoshida M, Inoue M, Iwasaki M, Tsugane S; JPHC Study Group. Association of body mass index with risk of age-related cataracts in a middle-aged Japanese population: the JPHC Study. Environ Health Prev Med. 2010 Nov;15(6):367-73.

Overall, I found this an interesting study, but believe that a deeper analysis, as outlined below, might substantially increase the impact of the work.
Abstract
2). Ors are shown for group comparisons, but odds ratios cannot be interpreted unless the scales of the variables involved is clear. Please clarify the scales. It looks like age is categorised whereas the DBQ scales are simply the item means or totals? This needs to be clarified. Standardising the DBQ scales might provide a more interpretable metric.
Response: Thank you for this comment. We clarified the cut-offs for violations, errors, and lapses in the abstract section as suggested (Page 2).
3). The OR for errors and lapses is written as if the difference between younger and middle-aged is significant but the CI on the OR includes 1. It needs to be clearer that this difference is ns, or perhaps left out of the abstract. This issue is also relevant in the discussion.

Response: We rephrased the abstract to differentiate between the absolute statistically significant associations and the tendency of some factors to be associated with aberrant driving behaviors (Page 2).
Introduction
4). P1 Older drivers are argued to be at risk of injuries due to cognitive decline. This is plausible, but there is work that provides an alternative explanation simply based on lower mileage. I recommend considering this paper in the consideration of this issue:
Langford, J., Methorst, R., & Hakamies-Blomqvist, L. (2006). Older drivers do not have a high crash risk—A replication of low mileage bias. Accident Analysis & Prevention, 38(3), 574-578. doi:https://doi.org/10.1016/j.aap.2005.12.002
Response: Thank you. This study was very helpful. We added this explanation to the discussion part to justify our findings (Page 12 lines 183-189).
Methods
5). Please say a little more about how the Arabic version of the DBQ was obtained and which of the many versions of the DBQ the questionnaire was based on. It looks like the commonly made distinction between aggressive and non-aggressive violations was not made here. On what basis was this decision made?
Response: More descriptions were added to the Arabic version of DBQ (Page 8 lines 82-98 & Page 9 lines 99-102). We made this decision based on the low scores of reliability and factorial analyses shown in a previous study using the Arabic version of DBQ and dividing the behaviors into four aspects.

- Bener A, Ozkan T, Lajunen T. The driver behaviour questionnaire in Arab Gulf Countries: Qatar and United Arab Emirates. Accid Anal Prev, 2008; 40:1411-1417.

6). Analyses: Additional control for annual mileage would be helpful if that was recorded.
Response: Since we limited our study population to nonprofessional drivers and given the fact that the streets in Egypt, including Beni-Suef, are very crowded, we thought that using driving hours may be more realistic in the current situation that mileage. Thus, we adjusted our results for driving hours.
Results
7). As currently presented, the results are interesting, and with note of the point about metrics made regarding the abstract, can be interpreted.
Response: Thank you. The abstract was modified as suggested in the first comment.
8). I would find a more detailed analysis of the DBQ data more interesting. I don’t think it can be assumed that the factor structure of the DBQ is stable across cultures. Therefore, the paper would benefit from discussion of this issue in the introduction and from analysis in the present paper. This might be an Exploratory or Confirmatory analysis, depending on whether the literature review provides a strong expectation for the factor structure to be observed in this context.
Response: We further discussed the possible cultural differences across DBQ versions (Page 8 lines 82-98 & Page 9 lines 99-102).
9). Was crash involvement measured? A key question about applying measures of driving risk developed in HICs to LMICs is whether they maintain their validity in their central purpose; to identify propensity for crash involvement. Therefore analyses testing the relationship with crash involvement would be helpful and the sample size seems big enough to provide a meaningful test of this relationship. Finding a significant relationship with crash involvement would support the application of the DBQ in Egypt, but if it is not found then that would still be an interesting result for the research community. Coverage of this issue in the introduction would also be helpful. This needs to be raised as a limitation if crash involvement was not measured.
Response: We did not assess crash involvement for 2 reasons: 1- This would greatly undermine the response rate leading to a high possibility of non-response bias because most traffic crashes that do not include injuries or include minor injuries are not reported to the police, so participants would be afraid of getting in legal troubles. A recent study from Egypt on crash involvement showed a response rate of only 52% and non-respondents expressed their concerns regarding data security. 2- Since many traffic crashes are not reported, we would not have any method of verification. However, we added this point as a limitation (Page 13 lines 199-202).

- Arafa A, El-Setouhy M, Hirshon J. Driving behavior and road traffic crashes among professional and nonprofessional drivers in South Egypt. Int J Inj Contr Safety Promot, 2019; 26:372-378.

Discussion
10). Ensure the discussion focusses only on significant comparisons. The idea of self-monitoring in older drivers is interesting and plausible. However, I was unclear how this was supported by the data collected.

 Response: We limited the discussion to statistically significant associations as suggested (Page 10 lines 145-147 & Page 11 lines 148-150). Unfortunately, we did not assess self-monitoring, but previous studies suggested this explanation to justify the relatively safe driving of old people. Therefore, we cited these studies to explain our findings (Page 12 lines 176-182).

- Anstey K, Wood J, Lord S, Walker J. Cognitive, sensory and physical factors enabling driving safety in older adults. Clin Psychol Rev, 2005; 25:45-65.
- Conlon E, Rahaley N, Davis J. The influence of age-related health difficulties and attitudes toward driving on driving self-regulation in the baby boomer and older adult generations. Accid Anal Prev, 2017; 102:12-22.
- Molnar L, Eby D, Charlton J, Langford J, Koppel S, Marshall S, et al. Driving avoidance by older adults: is it always self-regulation? Accid Anal Prev, 2013; 57:96-104.
