# Supplementary material for: Age-related differences in driving behaviors among non-professional drivers in Egypt
Source: PLoS One. 2020 Sep 3;15(9):e0238516. doi: 10.1371/journal.pone.0238516 (PMC7470271; doi:10.1371/journal.pone.0238516)
Supplement: Attachment — Submitted filename: Response 2 (PLOS ONE).docx [file pone.0238516.s003.docx]

Dear editor-in-chief,

Thank you for the favorable response.

Herein, our response to comments of reviewer 2

1. Abstract
   1.1. I am not convinced the sampling is random - were these volunteers? If so, volunteering is not random, so this needs to be clarified.

Response: Thank you for this comment. The sample was randomly selected. The selection process was described in detail (lines 72-81). Since data were self-reported and given the fact that we offered no incentives for participation, it could be speculated that different forms of bias could be present, and we already cited these limitations (lines 191-194).

1.2. Results- I still find the ORs difficult to interpret. I recommend removing the Ors from the abstract an just describing the significant associations in the data. I do not believe any interpretation should be given for non-significant associations, anywhere in the ms.

Response: We modified the results in the abstract section as suggested (page 3).

2. Introduction
   2.1. Now provides a solid introduction to the work in my reading.

Response: Thank you.

3. Method
   3.1. I don’t see the authors have dichotomised the DBQ scales to identify aberrant driving. The DBQ provides continuous measures of driving constructs and dichotomising them reduces the power of analysis without any benefit so far as I can see. I recommend analyses are re-run using the continuous DBQ scores as the outcome variables. This may mean that some of the tendencies identified in the data but falling below significance will become significant.

Response: While we appreciate your opinion, we have some concerns regarding treating DBQs scales as continuous scales rather than dichotomizing them for the following reasons:

1. This study aims, in the first place, to draw policies for traffic measures in South Egypt, therefore, the emerging results will be presented to officials and academics who might carry limited or no expertise in statistical analyses. We believe that presenting the results in the form of categorical variables will be easier to understand and can deliver our message directly. It is more convenient to claim that the younger age may be associated with a 46% increase in the risk of driving violations compared with the middle-aged than claiming that transition from middle age category to young age category may lead to an increase by 0.39 units on the violation scale of driving behaviors.
2. In response to your comment, we already re-ran the analysis, however, the results did not materially change.
3. In case of conducting linear regression to compute beta coefficients and their 95% CIs for violations, errors, and lapses, we will face another problem that other driving habits (using seatbelts, cellphones, eating, driving while feeling drowsy, and driving while feeling sleepy) are not continuous variables and conducting logistic regression in their case is a must. So, in such a case, the upper half of the table will be conducted by linear regression and presented by unit change and the lower half of the table will be conducted by logistic regression and presented by odds. Also, the descriptive data in the upper half will be presented by mean and Sd or median and IQR while the lower half will be presented by frequencies and percentages. We think that this inconsistency may leave the reader confused.
4. Discussion
   4.1. No interpretation or consideration should be given to ns results.

Response: We modified this point as suggested.

4.2. P12Line 176. The point about self-monitoring older drivers needs to be grounded in literature.

Response: This part is cited in references 29, 30, and 31.
