# Supplementary material for: Age-related differences in driving behaviors among non-professional drivers in Egypt
Source: PLoS One. 2020 Sep 3;15(9):e0238516. doi: 10.1371/journal.pone.0238516 (PMC7470271; doi:10.1371/journal.pone.0238516)
Supplement: Attachment — Submitted filename: Response (PLOS ONE 3).docx [file pone.0238516.s004.docx]

Reviewer #2: Many thanks for your responses. Most are very helpful, but I do believe the ms will be much more valuable if the DBQ is treated as a continuous measure.

I can see the advantages of dichotomising the DBQ scales for ease of communication to non-academic audiences. However, it is an oversimplification that may mislead. There is no evidence that the cutpoints you have identified are in anyway meaningful in terms of risk of crash, but if you present the results in this way, any audience, particularly an uninformed one, is likely to think that they do mean something.

The audience you are writing for at PLOS ONE are academics who are familiar with interpreting continuous statistics in general. The audience with expertise in driving behaviour will expect that the DBQ will be treated as a continuous measure as it has always been in the very many studies that I have authored and read using this instrument.

The problem of some analyses addressing dichotomous variables and some addressing continuous is a common one in writing research papers, so I have no doubt you will be able to find an unambiguous way to present your tables.

Response:

Thank you for your informative comment. We treated DBQ scales as continuous data as suggested and made some changes accordingly.

1- We divided table 2 to tables 2 and 3: table 2 for violence, errors, and lapses with associations conducted using linear regression and table 3 for driving habits such as eating, using the cell phone, and using the seatbelt during driving with associations conducted using logistic regression.
2- Some changes were made accordingly in the methods (lines 106-123), results (lines 130-140), and discussion sections (142-148).
